# Emergence of multiple fluorophores in individual cesium lead bromide nanocrystals

**DOI:** 10.1038/s41467-019-10870-1

**Published:** 2019-07-02

**Authors:** Yuhai Zhang, Tianle Guo, Haoze Yang, Riya Bose, Lingmei Liu, Jun Yin, Yu Han, Osman M. Bakr, Omar F. Mohammed, Anton V. Malko

**Affiliations:** 1 Division of Physical Sciences and Engineering, King Abdullah University of Science and Technology, Thuwal, 23955-6900 Saudi Arabia; 2 Institute for Advanced Interdisciplinary Research (iAIR), University of Jinan, Jinan, 250022 Shandong China; 3 Advanced Membranes and Porous Materials Center (AMPM), Division of Physical Science and Engineering, King Abdullah University of Science and Technology, Thuwal, 23955-6900 Saudi Arabia; 4 Department of Physics, The University of Texas at Dallas, Richardson, TX 75080 USA

**Keywords:** Solar cells, Nanophotonics and plasmonics, Nanoparticles, Organic-inorganic nanostructures, Quantum dots

## Abstract

Cesium-based perovskite nanocrystals (PNCs) possess alluring optical and electronic properties via compositional and structural versatility, tunable bandgap, high photoluminescence quantum yield and facile chemical synthesis. Despite the recent progress, origins of the photoluminescence emission in various types of PNCs remains unclear. Here, we study the photon emission from individual three-dimensional and zero-dimensional cesium lead bromide PNCs. Using photon antibunching and lifetime measurements, we demonstrate that emission statistics of both type of PNCs are akin to individual molecular fluorophores, rather than traditional semiconductor quantum dots. Aided by density functional modelling, we provide compelling evidence that green emission in zero-dimensional PNCs stems from exciton recombination at bromide vacancy centres within lead-halide octahedra, unrelated to external confinement. These findings provide key information about the nature of defect formation and the origin of emission in cesium lead halide perovskite materials, which foster their utilization in the emerging optoelectronic applications.

## Abstract (web summary)

Inorganic perovskite nanocrystals attract lots of research attention but the origin of their photoluminescence remains debatable. Here Zhang et al. show that behavior of both CsPbBr_3_ and Cs_4_PbBr_6_ nanocrystals is like individual molecular fluorophores and independent of the structural dimensionalities.

## Introduction

Unlike conventional chalcogenide nanocrystals (NCs) that are primarily composed of binary compounds, perovskite materials have predominantly ‘soft’ ionic lattice structure and typically crystalize into the quasi-cubic A^+^PbX^-^type structures (A^+^-methylammonium, formamidinium or cesium (Cs^+^), while X^-^ is a halide anion such as Cl^−^, Br^−^ or I^−^)^1–3^. Such structures can be conveniently depicted as comprised of lead halide octahedra [PbX_6_] surrounded with A cations, residing in the voids between them and arranged with varying degrees of interconnections. Among them, all inorganic Cs_n_PbX_m_ perovskites have attracted particular attention due to enhanced light emission and photo/thermal stability^4,5^. Lower dimensionality polymorphs can be formed by manipulation of chemical synthesis conditions where Cs^+^ can stabilize 3D [PbX_6_] framework, resulting in 2D (nanosheet), 1D (nanowire) and 0D (nanodot) internal octahedra arrays within the bulk of the perovskite^2^. The gamut of available experimental approaches are further expanded in colloidal perovskite NCs where both external size quantization and internal 0D structure may combine to achieve ‘multidimensional’ electronic properties that are engineered both on atomic scale and nanoscale.

Understandably, to fully exploit the potential of these perovskites NCs and to widely expand their light-conversion applications, one would need to obtain the precise knowledge of the origin and properties of their photoluminescence. Traditional semiconductor NCs that now find their way into a number of optoelectronic applications are comprising thousands of atoms organized in a bulk crystal structure^6^. In this particular case, it is well reported that size-dependent nature of optical transitions arises from the size quantization of a free motion of bulk electrons and holes within (typically) sub-10 nm, three- dimensional potential box^7^. Crystalline structure of high quality semiconductor NCs is essentially defect-free and they exhibit huge photoluminescence (PL) spectral shifts as a function of the size. Any reduction of photoluminescence quantum yield (PLQY) necessarily arises from inefficient surface passivation with organic ligands, which could form undesirable surface trap states. In a sharp contrast, Cs-based perovskite materials with their structural lability and much lesser degree of chemical stability are typically characterized by much higher density of point defects and, perhaps more importantly, they show very small (if any) PL spectral shift as a function of their size^8^. Nevertheless, many perovskite NCs are bright emitters, with PLQY close to unity^9^. Being in this regime, the unified picture of this defect formation along with the origin of the photon emission in these NCs has not yet been achieved.

**Fig. 1:**
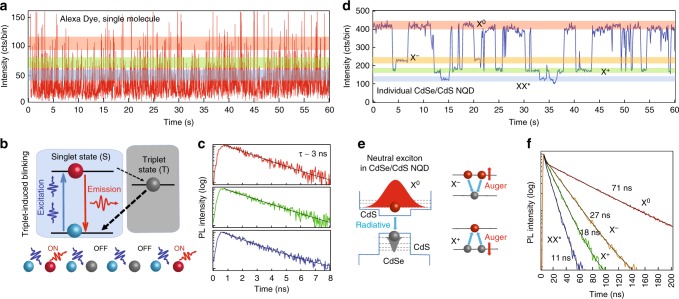
Representative blinking behavior of a molecular emitter and a colloidal nanocrystal quantum dot. **a**–**c** A single Alexa 594 dye molecule and **d**–**f** individual CdSe/CdS core/shell nanocrystal quantum dot. PL lifetimes extracted from different intensity levels are color-coded to highlighted regions in blinking traces. Molecular blinking is characterized by burst-like emission with nearly uniform distribution of lifetimes across the intensity range. NQD’s blinking shows well-defined emission intensity levels with discrete lifetime values due to radiative recombination in various excitonic complexes. X^0^—neutral exciton, X^−^ —negatively charged trion, X^+^—positively charged trion, XX*—multiply charged excitons or charged biexcitons. Bin size is 20 ms

Single photon statistical studies represent an ultimate approach not only to clarify the radiative and non-radiative carrier recombination pathways, but also to distinguish between semiconductor excitonic and molecular-like emission statistics^10^. More specifically, single-particle PL intensity trajectories, (commonly referred as PL blinking traces), provide a direct observation of the exciton and multiexciton states and their interaction with the environment. All individual fluorophores exhibit this phenomenon, ranging from single molecules^11^ to emissive vacancy centers^12,13^ to semiconductor nanocrystal quantum dots (NQDs)^14,15^. In brief, blinking appearance and mechanisms may often be classified into one of the two commonly (albeit not universally) occurring scenarios^10,16^. One, usual for molecular fluorophores and defect/vacancy centers, involves intersystem crossing between singlet-triplet molecular states facilitated by strong interaction with the environment. Molecular fluorophores typically harbor singly excited states (excitons). Notwithstanding a large body of research devoted to stabilization of single molecules in solid-state host matrices and proliferation of antioxidation agents such as Trolox^17,18^, the photostability remains questionable. It is also reported that PL intensity fluctuations of untreated single molecules often exhibit ‘burst-like’ behavior where the system mostly resides in the non-emissive, ‘OFF’ state and rarely transitions into the fluorescent, ‘ON’ state, with each state possessing distinctive PL lifetime^19,20^. Another scenario, common for semiconductor quantum-confined NQDs (CdSe/ZnS is a prime example), involves charge tunneling/ionization into surface trap states followed by non-radiative Auger recombination of the remaining charged exciton (trion), leaving only neutral excitons (X^0^) to emit photons. Featuring low density of surface states, colloidal NQDs with high PLQY often exhibit prolonged periods of high emission intensity due to uninterrupted exciton radiative recombination. Moreover, recently developed large core/large shell CdSe/CdS NQDs possess reduced Auger rates and exhibit radiative recombination from charged excitons and biexcitons^21,22^. Such blinking traces exhibit a number of distinct intensity levels corresponding to emission from various excitonic species which are identified by different PL lifetimes^23,24^. The representative examples of single molecule and NQD blinking are shown in Fig. 1.

In this work, we explore the photon emission statistics from individual 3D (CsPbBr_3_) and 0D (Cs_4_PbBr_6_) perovskite NCs in order to address both the origin and the behavior of their PL emission. Using time-correlated, time-stamped single photon counting (TCSPC), we obtain PL intensity trajectories and extract PL lifetimes and second-order correlation functions, *g*^2^(τ) at different excitation levels. Blinking traces show ‘burst-like’ intensity behavior, with large bin-to-bin fluctuations, similar to molecular fluorophores. PL lifetimes extracted from the sub-100 ms burst intervals are between 8 and 11 ns for all studied PNCs regardless of excitation levels. Recorded single photon emission statistics indicate that some of the measured PNCs are single photon emitters, others contain several emissive centers with very similar lifetimes. Few of the PNCs exhibited effects of photobrightening—superlinear increase of PL emission intensity due to the activation of an additional number of emissive centers within the PNC. Such emission behavior, independent of the confinement effects afforded by quantization in the medium/large sized Cs-based PNCs, supports theoretical framework that points towards emissive Br-vacancy states localized within isolated octahedra.

## Results

### Chemical synthesis of PNCs and their optical properties

**Fig. 2:**
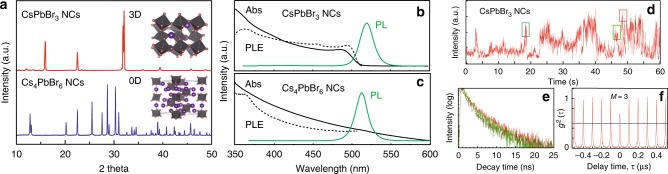
Structural and optical comparison between 3D CsPbBr_3_ NCs and 0D Cs_4_PbBr_6_ NCs. **a** XRD patterns of perovskite nanocrystals, with inset schematics showing a corner-sharing octahedra network and a discrete octahedra lattice, respectively. Room-temperature absorption (Abs, solid line), photoluminescence excitation (PLE, emission monitored at 520 nm, dotted line), and photoluminescence (PL, excitation at 350 nm, green line) spectra of **b** colloidal CsPbBr_3_ NCs and **c** Cs_4_PbBr_6_ NCs, respectively. **d** Blinking trace recorded for an individual CsPbBr_3_ NC at the excitation level of <*N*> = 1. **e** Extracted PL lifetimes from short, sub-500 ms bursts in a blinking trace and color-coded with highlighted regions in **d**. **f** Antibunching trace

3D (CsPbBr_3_) and 0D (Cs_4_PbBr_6_) perovskite NCs were synthesized through a reverse-microemulsion method at room temperature^25^. By controlling the feeding ratio of Cs/Pb precursor, the perovskite NCs of varied dimensionalities were obtained in an identical procedure, which affords an identical surface. The as-prepared 3D and 0D NCs show a similar narrow size distribution with an average diameter of 15 and 26 nm, respectively (Supplementary Fig. 1). X-ray diffraction (XRD) measurements suggest that 3D PNCs adapted an orthorhombic phase (P_nam_) with corner-sharing [PbBr_6_] octahedra while 0D PNCs adapted a rhombohedral phase (R_3c_) comprising isolated [PbBr_6_] octahedra bridged by Cs^+^ cations (Fig. 2a). It should be noted that distinctive XRD and HRTEM patterns clearly demonstrate the high-quality synthesis of pure-phase all-inorganic perovskite NCs, Supplementary Fig. 2. Absorption spectra of 0D PNCs, without any sign of excitonic peak, exhibit a long Urbach tail in the range from 350 nm to 600 nm, indicating a distribution of possible defect states in Cs_4_PbBr_6_ lattice (Fig. 2(b, c) and Supplementary Fig. 3). While their absorption and excitation profiles are rather different, PL emission spectra of both types of PNCs indicate remarkable similarities. The measured PLQY of 0D nanoparticles in solution routinely reached value of 60–70%. These values correspond well to a number of recent publications.

### Sample preparation and antibunching measurements

For both types of PNC samples (CsPbBr_3_—3D and Cs_4_PbBr_6_—0D), nanoparticles with low surface density were prepared by drop casting on quartz substrates from PMMA solutions. Using high numerical aperture confocal microscope system, individual PNCs were visualized and time-resolved PL signatures recorded using time correlated, time-tagged single photon counting (TCSPC) collection. More details of the measurements are provided in the “Methods” section. We recorded PL blinking traces and extracted PL lifetimes and second-order photon correlation functions (*g*^2^(τ), antibunching trace). At a low excitation level, when the probability to simultaneously excite two excitons (bi-exciton) per NC is negligible, it takes nonzero time for a quantum-mechanical system to cycle between ground and excited states. Hence, the probability to instantaneously detect two photons at zero delay time is reduced and the number of independent emitters within an individual PNC is calculated through the well-known statistical approach as *g*^2^(0) = 1–1/*M*, where *M* is the number of emitters^26^. Consequently, zero value of the antibunching function has been the distinctive feature of a single quantum emitter (*g*^2^(0) = 0 for *M* = 1) while values above 0.5 indicate multiple emitters (*g*^2^(0) = 0.5 for *M* = 2; *g*^2^(0) = 0.67 for *M* = 3, etc.)^27^. The average number of excitons <*N*> per PNC was computed using well-known expression <*N>* = *jσ*, where *j* is the number of incident photons per cm^2^ per pulse, and *σ* is the absorption cross-section of an individual NC, computed using Beer-Lambert law as shown in “Methods” section (additional information in Supplementary Fig. 4 and Supplementary Table 1).

### Single-particle measurements of 3D CsPbBr_3_ perovskite NCs

In our systematic approach, we first explored individual 3D CsPbBr_3_ NCs. In general, we found that at low excitation levels <*N*> = 0.1 to 0.2, the majority of 3D nanoparticles are single quantum emitters with low *g*^2^(0) values, consistent with a number of previous publications^28,29^. Extracted PL emission lifetimes are fitted with bi-exponential functions with τ_1_ approximately 2 to 4 ns and τ_2_ of approximately 8 to 11 ns. The two lifetimes stem from the contributions of two emissive states: longer lifetime corresponds to the emission from the neutral exciton (X^0^), while the shorter decay time could indicate emission from a charged exciton (X^−^, trion). Since the lifetime values are sufficiently close, monoexponential fitting often provides average lifetimes of τ_av_ of approximately 6 to 7 ns, consistent with the previous measurements^30,31^. A representative data set is presented in the Supplementary Fig. 5.

However, blinking behavior of some of the 3D CsPbBr_3_ NCs visibly changed at higher excitation levels. Figure 2d–f presents blinking trace, extracted lifetimes and antibunching at excitation level of <*N*> = 1. It is clearly seen that blinking trace is no longer represented by two-state emission and is now more ‘burst-like’, with contributions by *M*~3 emissive centers as indicated by the antibunching function. At the same time, the PL lifetimes of different bursts indicated by colored boxes in the blinking trace are very close (τ_1_ of approximately 2 ns and τ_2_ of 7 to 8 ns), suggesting their similar origins.

### Single-particle measurements of 0D Cs_4_PbBr_6_ perovskite NCs

**Fig. 3:**
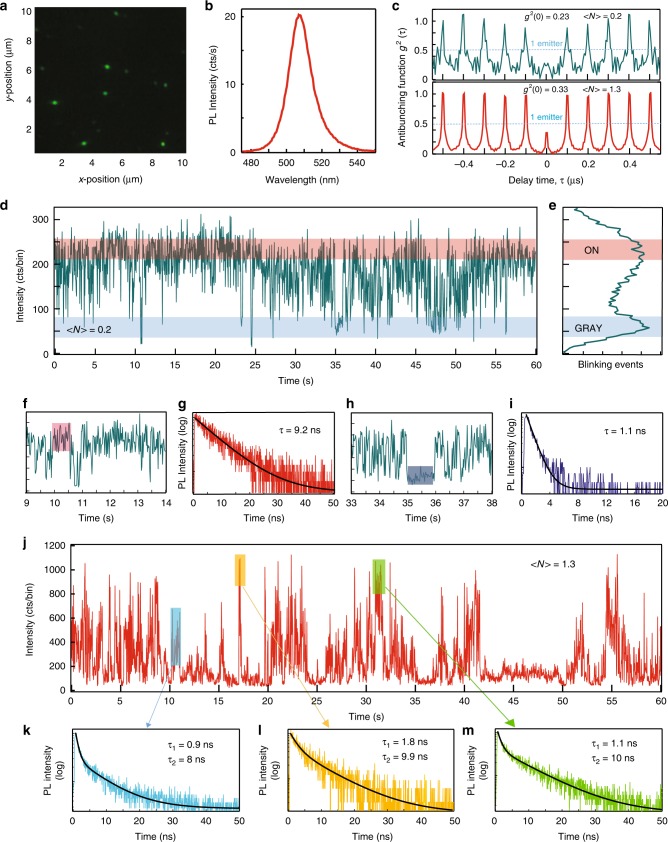
Blinking statistics of an individual 0D PNC at low **a**–**i** and high **j**–**m** excitation fluxes, respectively. **a** Wide-field illumination image of isolated 0D PNCs, excitation wavelength 400 nm. **b** PL spectrum of an individual 0D PNC. **c** Antibunching functions at different excitation levels, both show single emitter behavior (*g*^2^(0) <0.5). **d**, **e** Blinking trace and intensity distribution histogram at <*N*> = 0.2 with ‘ON’ and ‘GRAY’ states. **f**, **g** Expanded view of the blinking trace and PL lifetime extracted from the sub-second ‘ON’ intensity level shown by shaded bar. **h**, **i** Same for sub-second ‘GRAY’ level. **j**–**m** Excitation level of <*N*> = 1.3. **j** Blinking trace exhibiting burst-like behavior. **k**–**m** PL lifetimes extracted from different sub-second intensity bursts and color-coded to shaded bars in (**j**). Bin size is 20 ms

At the moment, there have been no reports of single photon emission from Cs_4_PbBr_6_ NCs. Figure 3 presents detailed blinking statistics for an individual 0D Cs_4_PbBr_6_ PNC that contains one quantum emitter at different excitation levels. Wide-field illumination image in Fig. 3a shows a collection of fluorescence spots corresponding to individual 0D PNCs, with the intensities affected by various degrees of blinking. The PL spectrum of an individual PNC shown in Fig. 3b, with the linewidth of about 14 nm, is considerably smaller than 23 nm spectral bandwidth measured for ensemble of 0D PNCs in Fig. 2c. Antibunching functions *g*^2^(τ) shown in Fig. 3c are measured at several excitation intensities corresponding to the average of <*N>* = 0.2 and <*N>* = 1.3 excitons per PNC and indicate emission from a single quantum emitter at both excitation levels. The PL intensity trajectory taken at <*N>* = 0.2 in Fig. 3d shows considerable bin-to-bin intensity fluctuation; however more defined ‘ON’ and ‘GRAY’ levels are now visible as indicated in distribution histogram in Fig. 3e. It should be noted that the apparent ‘noisiness’ of the blinking traces is not contributed by the background noise as its level is on the order of 10–20 counts per bin since we are using specially prepared quartz substrates and low power excitation. Instead, this behavior comes from very fast (much shorter than binning time) switching between the emissive states. Whenever the particle resides in a given state for a longer time, the blinking trace (or parts of it) looks almost noise-free as can be seen in the example of the CdSe/CdS dot shown in Fig. 1d. As previously reported^24^, in order to limit simultaneous contribution from both emissive states, PL lifetimes were extracted from much shorter, sub-second time intervals, shaded boxes in Fig. 3f, h. These lifetimes, presented in Fig. 3g, i, are nearly monoexponential with corresponding lifetimes of τ_ON_ = 9 ns and τ_GRAY_ = 1 ns, respectively, attesting to their origin from well-defined emissive states.

As the excitation power increases further, the blinking behavior of the same 0D PNC visibly changes as seen in Fig. 3j. We observe a multitude of intensity spikes that closely resemble the blinking behavior of individual molecular emitters. However, PL lifetimes extracted from different intensity bursts (shaded boxes) and shown in Fig. 3k–m are still composed of the same two components (1 ns and 9 to 10 ns) with varying amplitudes. Longer components predominate in the higher intensity bursts and the shorter ones in the low intensity intervals, consistent with the contributions of ‘ON’ and ‘GRAY’ states.

**Fig. 4:**
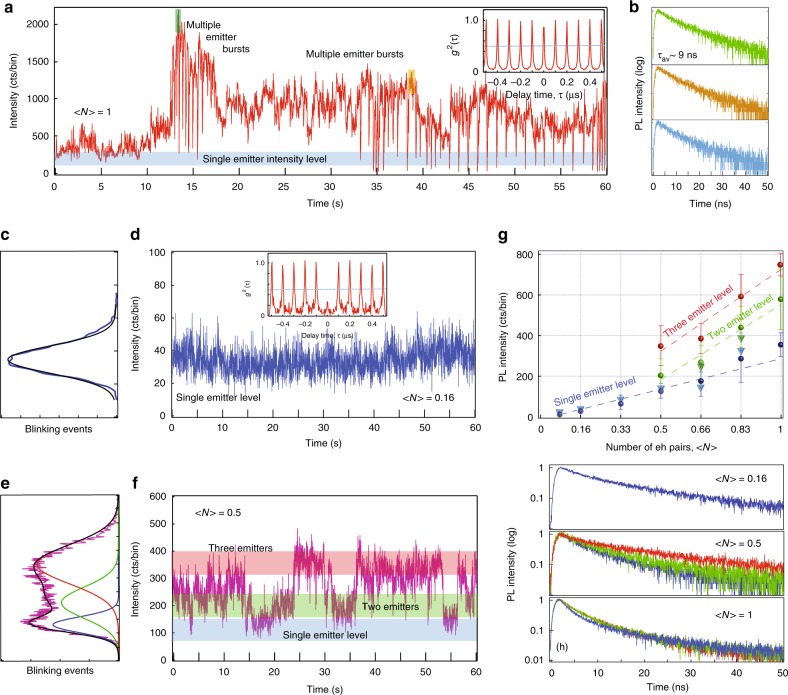
Blinking in two individual 0D PNCs (**a**, **b**) and (**c**–**h**) with the emergence of multiple emitters. **a** Blinking trace exhibiting large bursts from multiple emitters. Inset—antibunching trace with large *g*^2^(0) value. **b** PL lifetimes extracted from different sub-second intensity bursts and color-coded to shaded boxes or bars in (**a**). Excitation level <*N>* = 1. **c** PL intensity histogram and **d** blinking trace for another dot at low excitation level of <*N>* = 0.16. Inset—antibunching trace indicative of single emitter. **e** Intensity histogram for the same dot at <*N>* = 0.5. Black trace—three Gaussian fit, colored traces—decomposition to individual intensity levels. **f** Blinking trace with several emitter levels highlighted by colored bars. **g** PL intensity of each emission level as function of the excitation <*N>*. Solid dots correspond to the increase of excitation to <*N>* = 1, triangles—decrease to <*N>* = 0.08. Dotted lines—linear fits. Error bars correspond to the widths of the Gaussian distributions in intensity histograms. **h** PL lifetimes at several intensity levels and color-coded to shaded bars in the corresponding blinking panels. Bin size is 20 ms

Out of about 60 0D PNCs that we had measured using TCSPC method, many have shown single emitter behavior at low powers that often transitions to ‘bursting’ behavior at higher excitation levels (more data in Supplementary Fig. 6). However, unlike semiconductor CdSe/CdS dots, perovskite NCs photobleach much more easily at higher excitation levels. For those that withstand higher fluxes for longer times (about 10 to 15%), many have shown a very peculiar emission behavior. Figure 4a shows a blinking trace of a 0D PNC at <*N>* = 1 excitation level. It starts at a certain intensity level (300 to 400 counts per bin), typical for a single emitter at this fluence (compare to Fig. 3). After several seconds, the PL intensity starts to exhibit large jumps to the level of about 1000 to 2000 counts per bin in well-pronounced bursts that go back and forth many times. Amazingly, despite nearly an order of magnitude change in the PL signal, the lifetimes extracted from individual bursts (green and yellow colored boxes in the blinking trace) and from the single emitter level (blue shadowed bar) are very similar, τ_av_ = 9 ns, Fig. 4b. Compiled lifetime statistics of all bursts shown in Supplementary Fig. 7 further confirm this behavior. Such behavior would have been completely impossible for any multiexcitons that would show dramatically different lifetimes at lower intensity levels (i.e., will not exhibit any order of magnitude, upward ‘bursts’). As indicated by large value of the antibunching function (inset to Fig. 4a), this behavior is consistent with the appearance of multiple emitters. To quantify bursts’ appearance, we performed power dependent measurements of another PNC as seen in Fig. 4c–h (the previous dot photobleached in less than 10 min). It exhibits single emitter character at low excitation power as seen from nearly Gaussian distribution of the blinking intensity and photon antibunching, Fig. 4c, d. It, however, clearly develops three intensity levels already at <*N>* = 0.5, thus excluding any possibility of biexciton generation, Fig. 4e, f. Further, the PL signals of the newly appearing emitters (green and red shaded bars/peaks in the intensity distribution histogram) appear much above the single emitter level. Figure 4g shows PL intensity *vs*. excitation power for single emitter (blue dots), two emitters (green dots) and three emitters (red dots). Single emitter level scales linearly with the excitation power with multiple emitter levels appearing clearly above. Hence, the total emission intensity grows superlinearly, in accordance with the increased number of emitters that contribute to the signal. Finally, the PL lifetimes extracted from each of the emitter levels stay nearly constant, indicating close similarity for the emitters at different intensity levels, Fig. 4h. Full set of blinking traces and corresponding intensity distribution histograms at all power levels is presented in Supplementary Fig. 8.

## Discussion

The PL emission statistics observed in 0D perovskite NCs is distinctively different from those of typical quantum-confined particles such as semiconductor quantum dots. Individual NQDs are single emitters with very low values of *g*^2^(0). Blinking behavior and antibunching values remain nearly unchanged at higher excitation powers due to Auger quenching of higher order excitations^32^. In some of the recently developed CdSe/CdS “giant” NQDs, *g*^2^(0) values may range between 0 and 1 depending on the radiative quantum yield of biexciton recombination that leads to nearly simultaneous emission of two photons from the same NQD^33^. However, their blinking traces always exhibit pronounced power dependence, with a number of well-identified intensity levels corresponding to the emission from various excitonic species, all characterized by different PL lifetimes as clearly seen in the example in Fig. 1. Furthermore, in our experiments with 0D PNCs we purposely kept number of excitons <*N*> low enough to largely avoid biexciton generation and cross-checked our *g*^2^(0) results using the time-gated technique that excludes photons generated in quick succession as for biexciton-exciton cascade^34^.

Thus, the superlinear increase of emission intensity concurrent with the appearance of burst-like blinking trajectories characterized by uniform PL lifetime distributions and increased values of *g*^2^(0) can only be explained by the appearance of additional emissive centers. This implies photoactivation of a number of emitters that act independently of each other. Recent publication has reported intensity photoactivation behavior in 3D CsPbBr_3_ and CsPbBr_2_I bulk PNCs samples and suggested structural reorganization of the perovskite matrix and filling of the trap states as possible causes of the light-induced changes^35^. The report, however, was not able to address emission changes at the level of individual PNCs, hence missing out important differences. On the other hand, it has been previously reported that the passivation of surface traps by photoadsorbed molecules^36^, photoinduced rearrangement of surface stabilizing agents^37^, and photoinduced neutralization of local charged centers^38^ are possible mechanisms of PL enhancement in semiconductor NQDs. Being in this regime, ref. ^39^ presents a comprehensive review of the photoactivation mechanisms in semiconductor NQDs. All of the above mechanisms have a common result—they remove surface defects and correspondingly enhance emission quantum yield for each QD in an ensemble. The confluence of these mechanisms leads to simultaneous increase of the emission intensity and intrinsic PL lifetimes in accordance with improved PLQY. However, photoactivation has not been reported to induce the appearance of the new emissive centers within individual NQDs. The same mechanisms may be at work in 0D PNCs, but lead to the enhancement of QY for the localized emitters situated well inside the individual PNC, causing simultaneous intensity photobrightening and observation of the increased number of emissive centers with similar emission parameters. It is quite notable that photoactivation behavior observed in our experiment (triangles in Fig. 4(g)) is reversible as can be seen by the convergence of the PL emission back to the single intensity level as the excitation power is decreased.

**Fig. 5:**
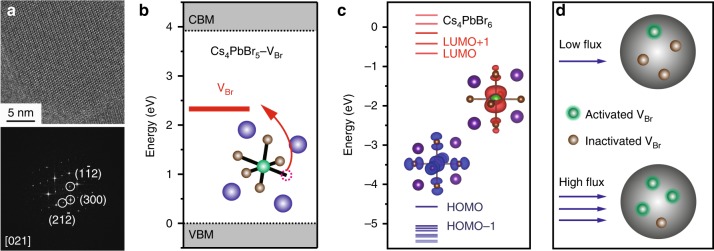
Experimental and theoretical explanation of the Br-vacancy origin of PL in 0D Cs_4_PbBr_6_ NC. **a** HRTEM image and corresponding FFT pattern of a single Cs_4_PbBr_6_ PNC along the [021] zone axis, showing clear lattice fringes without any impurity phase. **b** DFT calculation of *V*_Br_-induced mid-gap state between conduction band minima (CBM) and valence band maxima (VBM) of Cs_4_PbBr_6_ crystal, **c** DFT calculation of molecular energy levels of a single Cs_4_PbBr_6_ unit, insets show a highly localized density of states of LUMO level. Note that both DFTs were performed at HSE + SOC level. **d** Schematics of the activated/inactivated Br vacancies at different excitation powers

Considering the multitude of the experimental facts, we conclude that PL emission in our 0D PNCs (and possibly in the large/medium size 3D PNCs) is not affected by the nanocrystal’s external confinement factors. Instead, we conjecture that PL is the result of the carriers recombination at vacancy centers within [PbBr_6_]^4−^ octahedral. Several reports argue that 3D-phase impurity could be possible origin for the green emission in 0D PNCs^40^. However, our 0D samples have high PLQY, 60 to 70%. Their absorption spectra are very different from non-emissive 0D PNCs (Supplementary Fig. 3a), and also of 3D PNCs. In this work, since Cs_4_PbBr_6_ PNCs have a labile nature to electron beam, low-dose atomic-resolution TEM was used to examine the purity of the emissive Cs_4_PbBr_6_ PNCs at sub-lattice level. Our results show a clean lattice without any signal of impurity phase as shown in Fig. 5a and Supplementary Fig. 2. Further, to exclude the possibility of 3D phase formation induced by the laser exposure we have performed characterization measurements before and after UV illumination. Ensemble XRD (Supplementary Fig. 9) show identical spectra. Finally, we did a statistical (albeit limited in scope) study by HRTEM imaging of randomly chosen individual PNCs before and after UV exposure and found no evidence for the formation of the 3D structure, Supplementary Fig. 10.

To prove the vacancy origin of green emission, we performed density functional theory (DFT) calculation first on a perfect Cs_4_PbBr_6_ crystal and then on an imperfect Cs_4_PbBr_6_ crystal with an induced Br vacancy^4,41^. The Heyd-Scuseria-Ernzerhof hybrid functional (HSE06) including spin-orbit coupling (SOC) was used to calculate the electronic band structures (Fig. 5b). The DFT calculation shows that perfect Cs_4_PbBr_6_ crystal has an intrinsic direct bandgap of 3.9 eV, which is in line with the experimental data on non-emissive Cs_4_PbBr_6_ PNCs. Upon introducing a Br vacancy into the lattice, a mid-gap state immediately emerges at 2.4 eV, which is in good agreement to the PL energy of our emissive PNCs, qualifying Br-vacancy a strong candidate as the emitting center in Cs_4_PbBr_6_ PNCs^4,42^. Experimentally, we purposely reduced the concentration of the Br precursor to induce more Br vacancies and observed the increase of green PL emission, justifying this point^4^.

The blinking statistics, together with the large binding energy of Cs_4_PbBr_6_ PNC point to a highly localized nature of excitons, consistent with the molecular-like behavior of PL emission. In fact, the [PbBr_6_]^4−^ octahedra in 0D perovskite can, indeed, be viewed as an array of discrete molecules due to their isolated nature^41^. To prove this point, we performed DFT calculation on a single molecule of Cs_4_PbBr_6_ (Fig. 5c). The calculation generates an identical bandgap value (3.9 eV) to that of Cs_4_PbBr_6_ crystal, validating the molecular model of Cs_4_PbBr_6_. Under such a model, similar PL lifetime values and photobrightening effects result from photoactivated Br vacancies that act as individual single molecular emitters, Fig. 5d. In our experiments, we also observed that few of 0D PNCs exhibited multiple emitters’ behavior even at the low excitation power (Supplementary Fig. 7), indicating that some of these defects are active from the beginning, while others may be photoactivated, Fig. 5d. In fact, a recent publication has shown the emergence of strong luminescence bands upon gamma irradiation of Cs_4_EuBr_6_ and Cs_4_EuI_6_ 0D nanocrystals and identified the induced halide-related defects as the emission’s source^43^. Thus, we can suggest that laser-induced structural re-organization in 0D PNCs (and possibly in 3D) may lead to the formation of PL-active Br vacancy states. Having small lateral dimensions, these molecular-like states are not influenced by the quantum-confined effects whenever particle′s size is sufficiently large. However, strong confinement predicted for NCs with sizes of less than 5 nm may affect these states and shift their emission towards the higher energy as recently observed^1^. While gamma irradiation may have formed permanent defects, lower energy UV light may only induce quasi-static re-organization, as evidenced by reversibility seen in Fig. 4g. This behavior could possibly be related to the photoinduced ion migration as have been recently proposed^44,45^. We are continuing to study PL emission statistics at different experimental conditions to clarify these issues.

To summarize, we combined experimental and theoretical approaches to study 3D (CsPbBr_3_) and 0D (Cs_4_PbBr_6_) perovskite NCs at individual particle level. We demonstrated that at lower excitation levels both types of PNCs exhibited similar single photon emission behavior, independent of their structural dimensionalities (3D versus 0D) and nanocrystal’s size. Contrary to conventional metal-chalcogenide NQDs, an increase in excitation power induced the appearance of burst-like emission behavior with uniform distribution of PL lifetime values. A subset of 0D PNCs exhibited a photobrightening effect due to the photoactivation of several emissive centers within the same PNC. Overall, the experimental data provide a compelling evidence that emission from both types of PNCs closely resembles that of the individual single molecules and is independent on the external confinement factors. Aided by DFT calculations, we identified green PL emission in Cs_4_PbBr_6_ PNCs stemming from the exciton recombination at Br vacancy sites within [PbBr_6_]^4−^ octahedra. This systematic study of light emission statistics provides vital insights into the nature of defects formation and the decisive role they play in the origin of PL in cesium lead halide perovskite nanocrystals.

## Methods

### Materials

All reagents were used without any purification: Cs_2_CO_3_ (cesium carbonate, 99%, Sigma-Aldrich), OA (oleic acid, 90%, Sigma-Aldrich), OLA (oleylamine, 90%, Sigma-Aldrich), DMF (N,N-dimethylformamide, 99.8%, Sigma-Aldrich), 1,2-dichlorobenzene (DCB, anhydrous, 99%, Sigma-Aldrich) and anhydrous n-hexane (99.98%, Sigma-Aldrich).

### Synthesis of Cs_4_PbBr_6_ and CsPbBr_3_ nanocrystals

Cs_4_PbBr_6_ nanocrystals were synthesized using a modified reverse microemulsion method^46^. In a typical procedure, the PbBr_2_ precursor and the Cs-oleate precursor were synthesized separately. First, a mixture of 2.25 g of Cs_2_CO_3_ and 21.5 mL of OA was stirred and degassed at 130 °C under vacuum for 1 h to generate a transparent stock of Cs-oleate precursor. Second, 0.2 mL Cs-oleate precursor, 10 mL n-hexane, 5 mL OA were loaded in a 20-mL glass vial. Third, into the flask, a well-mixed (by vortex) solution of PbBr2 (0.03 M, DMF, 1 mL), HBr (48 wt%, 15 μL), 0.1 mL OA, and 0.05 mL OLA was swiftly injected under vigorous stirring. A color change from pale-white to pale-green was observed in 10 min, suggesting the formation of Cs_4_PbBr_6_ nanocrystals. The as-synthesized nanocrystals were collected via centrifugation at 8000 rpm for 3 min. The pellet was rinsed with 1 mL of DCB, followed by dispersion in 2 mL of DCB for further characterization. CsPbBr_3_ nanocrystals were synthesized using a similar method as above, except for the usage amount of PbBr2 precursor (0.24 M, DMF, 1 mL).

### Steady-state measurements of photoluminescence and absorption

The nanocrystals were diluted 100 times in n-hexane for steady-state measurements of photoluminescence and absorption, respectively. A Cary 5000 UV-vis spectrometer (Agilent Technologies) was used for absorption measurements in the range from 250 nm to 600 nm. A FluoroMax-4 spectrofluorometer (Horiba Scientific; slit width of 2 nm and scan rate of 500 nm/min) was used to record the photoluminescence spectra. The excitation wavelength used for the nanocrystals were set at 350 nm.

### X-ray diffraction measurements

Powder X-ray diffraction was performed using a Bruker AXS D8 diffractometer with Cu-Kα radiation (λ = 1.5406 Å). The samples were prepared via the drop casting of the nanocrystal’s suspension onto a clean glass slide, followed by drying at room temperature. Note that the Cs_4_PbBr_6_ nanocrystals are highly hygroscopic and tend to decompose when dried in air. Therefore, we prepared thin-film samples in a glovebox.

### Preparation of single-particle sample on a quartz slide

Quartz slides (2 cm × 2 cm) were cleaned by soaking in NaOH-saturated ethanol solution for 12 h, followed by thorough rinsing with DI water and isopropanol, respectively. The clean slides were dried with nitrogen flow at room temperature. Then, 0.2 μL of as-prepared Cs_4_PbBr_6_ nanocrystals in DCB was dispersed in 1 mL of PMMA solution in chloroform (1 wt%) by vortex. 10 μL of the solution was drop cast on quartz slide in a glovebox. The dried sample was used for single-particle measurement.

### Single-particle spectroscopy

For single-particle spectroscopy, the sample is mounted on a translation stage of a home-made optical microscope and perovskite NCs are excited at 445 nm with approximately 70 ps pulses from a laser diode through a 100×, 1.4 NA oil-immersion objective that is also used to collect PL. The interpulse duration is set to be much longer than the PL decay times in order to ensure complete relaxation of excitations between sequential laser pulses. Filtered PL signal (long pass 480 nm and bandpass 520/40 filters were used to reject scattered laser light and isolate emission from PNCs) is sent to a pair of avalanche photodiodes (PDM series, Micro Photon Devices) positioned at two arms of the standard Hanbury−Brown−Twiss (HBT) arrangement with a 50/50 beam splitter. Time-tagged, time-correlated single photon counting (TCSPC) is performed using PicoQuant HydraHarp 400 electronics. TCSPC simultaneously records photon arrival times with respect to the beginning of the measurement cycle and to the excitation laser pulse. Hence, it allows us to compile PL decay curves for any particular time segment of the PL intensity trajectory or a chosen window of the intensity distribution function while simultaneously recording second-order photon correlation functions, *g*^2^(*τ*). The overall system’s time resolution was better than 300 ps.

### Transmission electron microscopy

TEM images of CsPbBr_3_ nanocrystals were acquired using a Tecnai transmission electron microscope with an acceleration voltage of 120 keV. It is worth noting that the Cs_4_PbBr_6_ nanocrystals were not as stable as CsPbBr_3_ nanocrystals under irradiation of electron beams. Low-dose HRTEM images were acquired by a Cs-corrected FEI G2 cubed Titan 60–300 electron microscope operated at 300 kV, which is equipped with a Gatan K2 Summit electron-counting camera. The setup was operated in electron-counting mode at 4 k × 4 k resolution) with an image output rate of 20 fps at 4 k × 4 k resolution. An exposure of 6 s, therefore, results in an image stack of 120 individual image frames. These frames can be summed to improve the signal-to-noise ratio (SNR).

## Supplementary information

Supplementary Information

## Data Availability

The data supporting the findings of this study are available from the corresponding authors upon request.
