# Supplementary material for: Emergence of multiple fluorophores in individual cesium lead bromide nanocrystals
Source: Nat Commun. 2019 Jul 2;10:2930. doi: 10.1038/s41467-019-10870-1 (PMC6606627; doi:10.1038/s41467-019-10870-1)
Supplement: Supplementary file 1 — Supplementary Information [file 41467_2019_10870_MOESM1_ESM.pdf]

Supporting Information

Emergence of Multiple Fluorophores in Individual Cesium Lead Bromide Nanocrystals

Yuhai Zhang[1,2], Tianle Guo[4], Haoze Yang[1], Riya Bose[4], Lingmei Liu[3], Jun Yin[1], Yu Han[3], Osman M. Bakr[3], Omar F. Mohammed[1]*, Anton V. Malko[4]*

[1]Division of Physical Sciences and Engineering, King Abdullah University of Science and Technology, Thuwal 23955-6900, Kingdom of Saudi Arabia.

[2]Institute for Advanced Interdisciplinary Research (iAIR), University of Jinan, Jinan 250022, Shandong, China.

[3]KAUST Catalysis Center, Division of Physical Sciences and Engineering, King Abdullah University of Science and Technology, Thuwal 23955-6900, Kingdom of Saudi Arabia.

[4]Department of Physics, The University of Texas at Dallas, Richardson, TX, 75080, USA

Supplementary Figures:

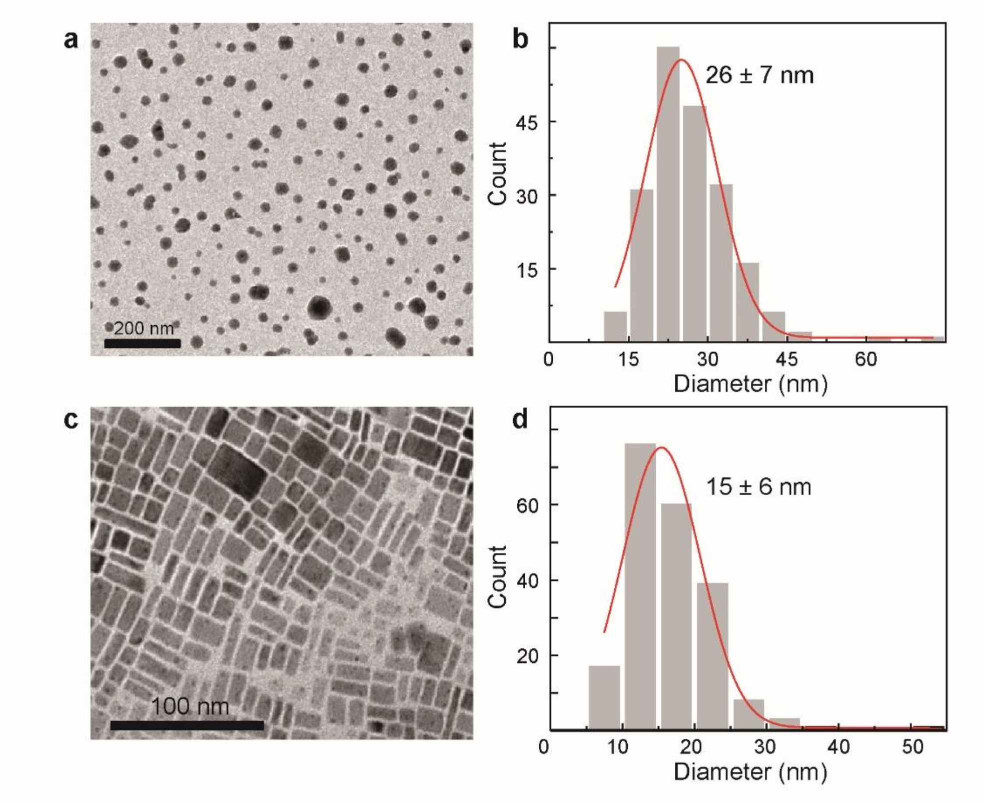

Supplementary Figure 1. TEM images and corresponding size histograms of (a, b) Cs_4PbBr_6 0D NCs and (c, d) $CsPbBr_3$ 3D nanocrystals, respectively.

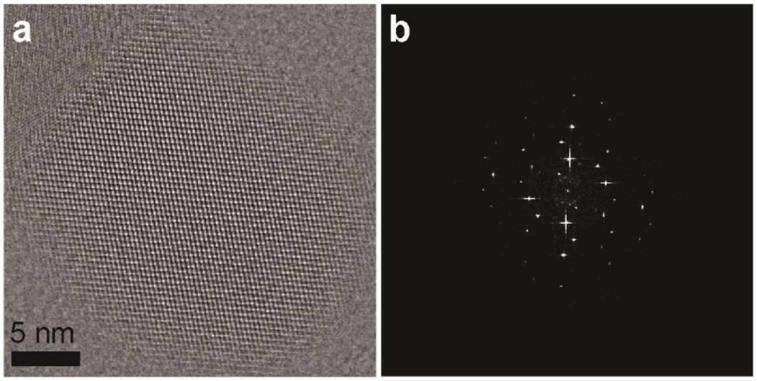

Supplementary Figure 2. HRTEM image and selected area diffraction pattern of a single 0D Cs_4PbBr_6 nanocrystal before UV illumination, showing the absence of any impurity inclusion.

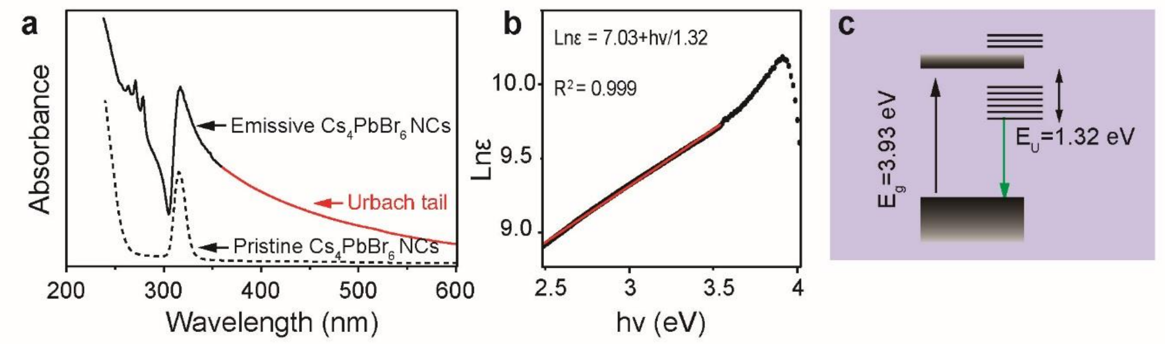

Supplementary Figure 3. (a) Absorption spectrum of emissive Cs_4PbBr_6 NCs and non-emissive (pristine) Cs_4PbBr_6 NCs[3]. Compared with non-emissive NCs, the emissive one shows a clear Urbach tail extended up to 600 nm, indicating the presence of defects or lattice disorder. (b) Urbach energy fitting in the range from 2.5 eV to 3.5 eV generates an Urbach energy of 1.32 eV, and the R^2 value validates the Urbach energy model[4]. (c) Schematic showing a possible energy diagram of emissive Cs_4PbBr_6 NCs.

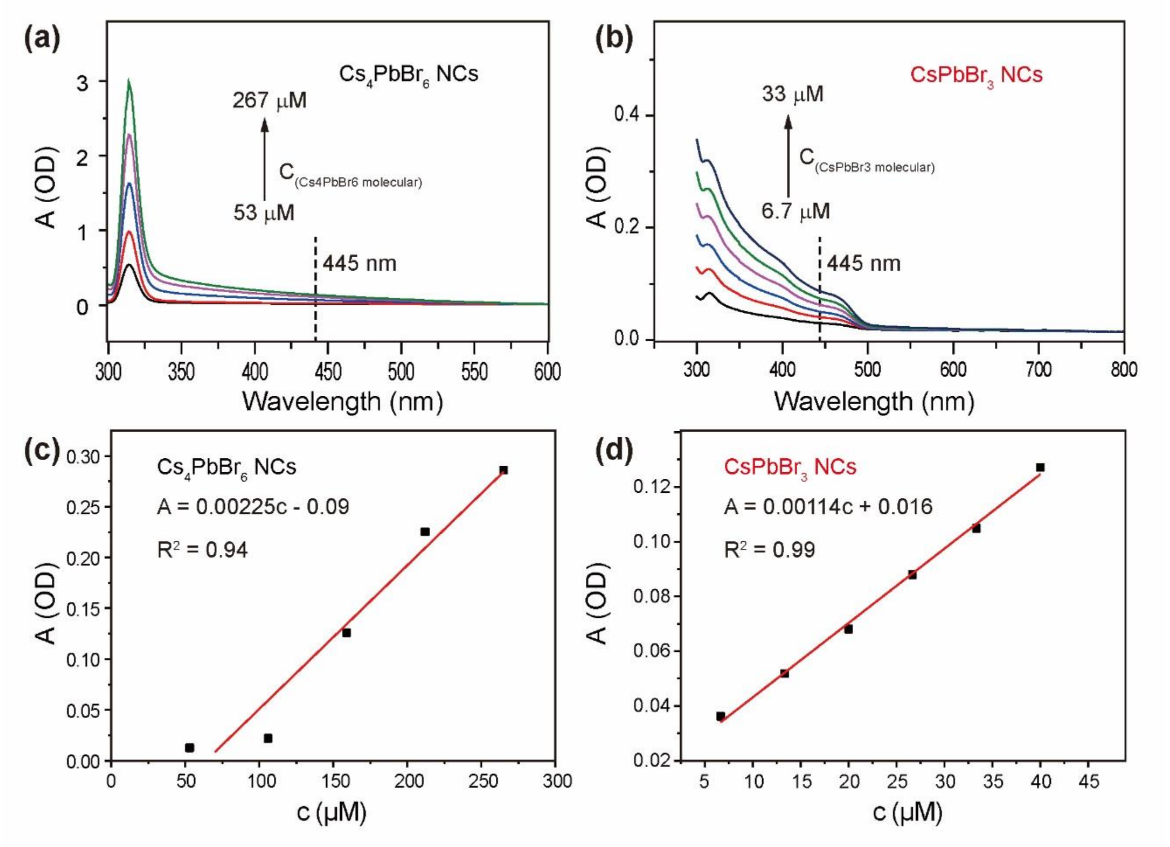

Supplementary Figure 4. Absorption spectra of Cs_4PbBr_6 NCs (a) and $CsPbBr_3$ NCs (b) as a function of varied concentration. (c, d) Absorbance at 445 nm were plotted against monomer concentrations, and the molar absorption coefficient of a monomer (Cs_4PbBr_6 or $CsPbBr_3$ molecule) can be obtained by Beer-Lambert law, that is, the slope of the linear fitting.

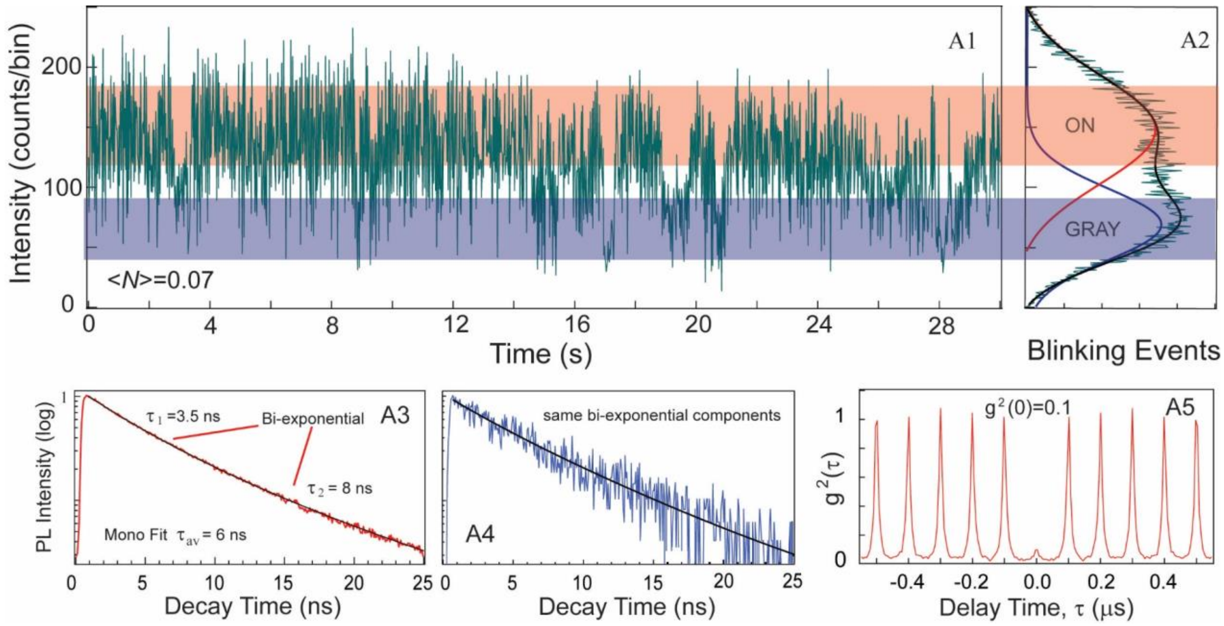

Supplementary Figure 5. Blinking in individual 3D PNC. (A1) Blinking trace. (A2) Intensity distribution histogram. Red and blue curves – Gaussian fits for 'ON' and 'GRAY' states, black curve – superposition of the both states. Bin size is 20 ms. (A3) Red trace - PL lifetime extracted from the pink-shaded intensity level recorded for the entire length of the intensity trajectory (average 'ON' lifetime). Black trace – bi-exponential fit. (A4) Blue trace - PL lifetime extracted from sub-100 ms short burst in the top intensity level, black line - bi-exponential fit with the same lifetime constants as in (A3). (A5) Antibunching trace. Excitation level <*N*> = 0.07

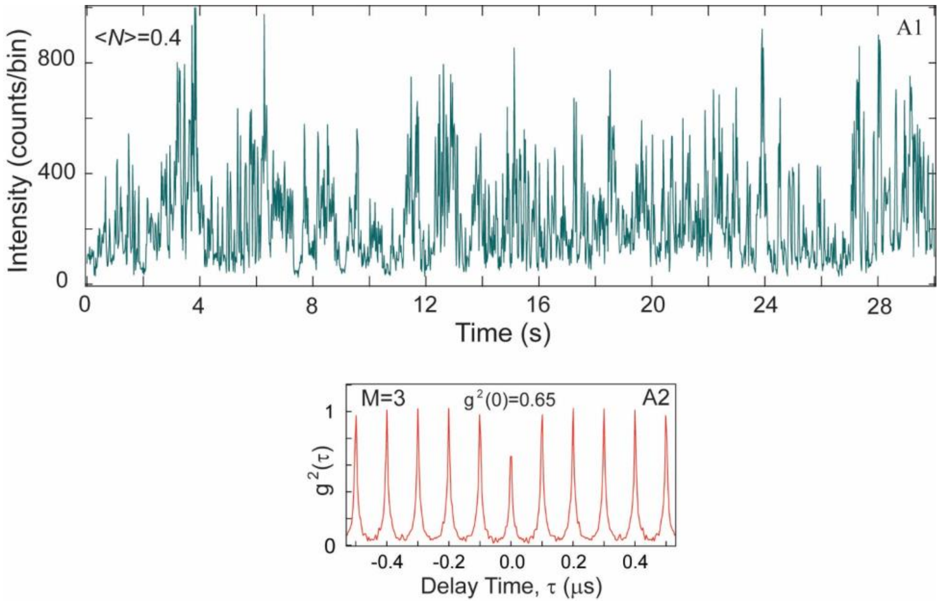

Supplementary Figure 6. Blinking in individual 0D PNC at low excitation level <*N*>=0.4. (A1) Blinking trace with multiple bursts. Bin size is 20 ms. (A2) Antibunching trace that shows presence of 3 emitters (M=3).

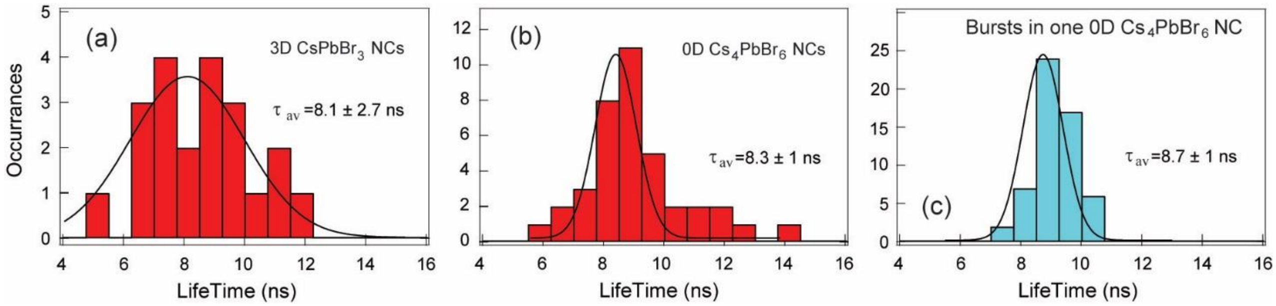

Supplementary Figure 7. (a, b) Lifetime distribution histograms collected for a number of PNCs that exhibit single quantum emitter behavior. (a) 3D nanocrystals (b) 0D nanocrystals. Lifetimes are extracted from short (100-500 ms) time intervals during 'ON' intensity level. (c) Lifetimes extracted from bursts recorded in the blinking trace of a single 0D PNC shown in the main text, Figure 4 (a).

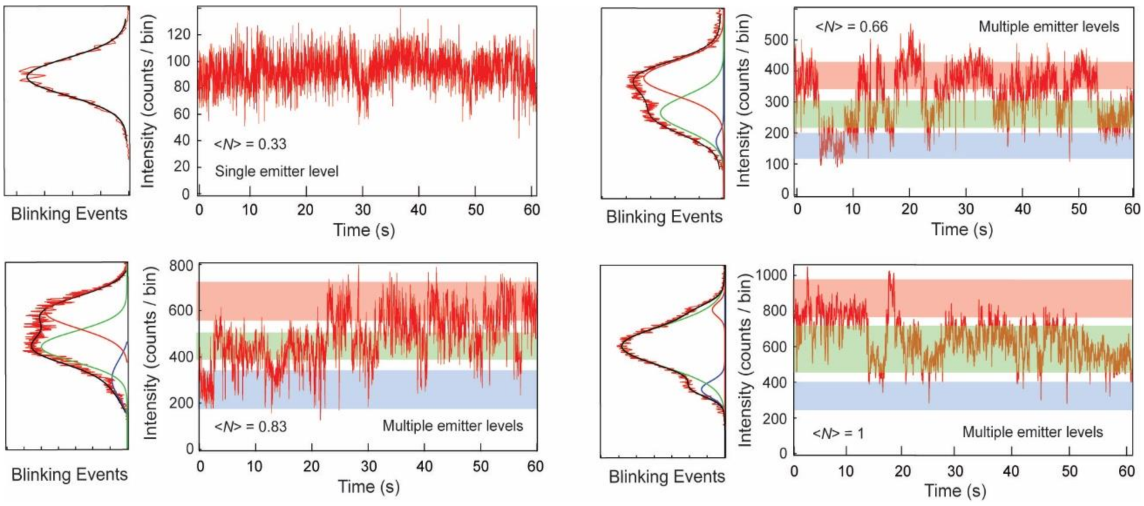

Supplementary Figure 8. Blinking and intensity distribution histograms at additional intensity levels used in the main text, Figure 4 (g), to extract multiple emitter behavior. Only 60 sec duration of blinking traces are shown for illustration. The intensity histograms were compiled from the entire duration of blinking traces, between 5-10 minutes.

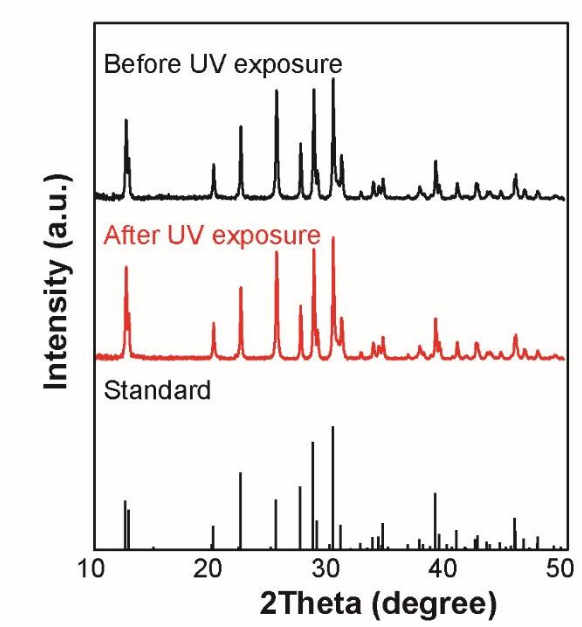

Supplementary Figure 9. XRD measurement of 0D PNCs before and after UV exposure, indicating that no phase transformation occurred during UV treatment.

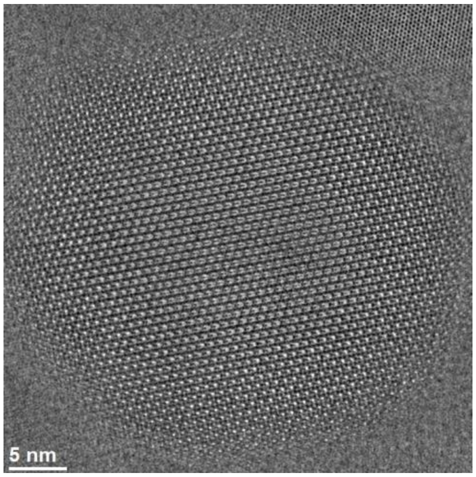

Supplementary Figure 10. HRTEM image of an individual 0D PNC after UV illumination. No evidence of 3D phase is found.

Supplementary Tables:

	$CsPbBr_3$ NC	Cs_4PbBr_6 NC
ρ (g cm^-3)	4.75	4.29
V_{NC} (cm^3)	1.19E-18	9.20E-18
Mw (g mol^-1)	5.80E+02	1.22E+03
ε (cm^2 mol^-1)	1.14E+06	2.25E+06
N	8.71E+03	1.95E+04
σ (cm^2)	1.11E-14	7.29E-14

Table 1. Useful information used in calculation of σ for $CsPbBr_3$ and Cs_4PbBr_6 nanocrystals. Note that *ε* was obtained by fitting absorbance data in Supplementary Figure 4, and V_{NC} was obtained by the using the size histogram in Supplementary Figure 1. Note that $CsPbBr_3$ has certain solubility in hexane where the absorption coefficient was measured, and using highly diluted colloidal solutions may lead to an overestimation of the $CsPbBr_3$ concentration and an ensuing underestimation of *ε*.

Supplementary Note 1:

Calculation of Absorption Cross-Section Per Particle (σ)

The molar absorption coefficient ε may be computed by using Beer- Lambert law (Supplementary Figure 4)[2]:

$$A = \varepsilon l c \qquad (1)$$

A is the absorbance of the sample defined as a logarithm of the ratio of the incident to transmitted power, *l* – optical pathlength, and *c* is the molar concentration of molecules/species. Hence, the molar absorption coefficient may be obtained as a linear slope of the absorbance vs. known concentration. It is worth noting that molar absorption coefficient is an intrinsic property of the specific molecules and is not affected by the size of particles. Then, the absorption cross-section per particle is proportional to the number of molecules found in the particle:[3]

$$\sigma = \frac{\varepsilon}{N_A} N \qquad (2)$$

σ is the absorption cross-section (cm^2) per particle, N_A is the Avogadro's number, N is the number of unit cells or molecules contained in a single particle which can be calculated from the following equation:

$$N = \frac{\rho V_{NC}}{M_w} N_A \qquad (3)$$

ρ is the mass density of particle's material, V_{NC} is the volume of a single nanocrystal particle, M_w is the molecular weight of a single unit cell comprising particle's lattice.

After combining equation (1) and (3) into (2), we obtain absorption cross-section σ as:

$$\sigma = \frac{\rho V_{NC} \varepsilon}{M_w} \qquad (4)$$

Related parameters and computed values of absorption cross-section can be found in **Supplementary Table 1**.

Supplementary Note 2:

Single particle measurements using 3D perovskite nanocrystals

Supplementary Figure 5, panel (A1) shows PL blinking trace of a 3D $CsPbBr_3$ nanocrystal that contains only one quantum emitter as confirmed by low value of $g^2(0)$ ~ 0.1 (panel A5). The intensity trajectory visibly appears to have a suppressed blinking behavior, *i.e.* without clearly defined "ON" and "OFF" intervals. However, closer look reveals that intensity fluctuates widely bin to bin (bin size is 20 ms). Intensity distribution histogram in panel (A2) shows a wide distribution, however it is still possible to fit with only 2 Gaussians corresponding to 'ON' and 'GRAY' states. A common way to analyze emission of individual chromophores is through analyses of PL lifetimes corresponding to different emissive states. Panel (A3) shows PL lifetime extracted from the pink-shaded intensity level recorded for the entire length of the intensity trajectory (average 'ON' lifetime). Monoexponential fit provides average value of τ_{av} ~ 6 ns, consistent with previous reports on single $CsPbBr_3$ nanocrystals. However, bi-exponential fit provides a better fidelity and returns values τ_1 ~ 3.5 ns and τ_2 ~ 8 ns. Commonly, the longer lifetime would correspond to the emission from the neutral exciton (X^0), while shorter decay time could indicate emission from a charged exciton (X^-, trion) Furthermore, PL lifetime extracted from sub-100 ms top intensity intervals and shown in panel (A4) is also bi-exponential with the same lifetime constants, but somewhat different amplitudes. Belonging to the 'ON' state, the lifetime value has larger amplitude of the long component that represents X^0 emission. The apparent 'fluctuating' behavior and superposition of short and long lifetimes at any macrotime scales (from sub-100 ms to minutes) corresponds to fast cycling between two emissive states within the same measurements bin(s).

Comparison of Single Particle Behavior in 3D and 0D PNCs

Statistical analysis of photon emission for several dozens of 3D and 0D nanocrystals in our study has shown remarkable similarities. Blinking traces for both types of PNCs at low excitation powers often exhibit 'quasi non-blinking' behavior due to the fast cycling between two emissive levels, while at higher powers they frequently show 'burst-like' intensity fluctuations. Supplementary Figure 6 shows statistical analysis of the PL lifetimes for both types of PNCs in the range of 7 to 11 ns, with larger variations observed for 3D PNCs.

Supplementary References:

1. Zhu, H.; Lin, C. C.; Luo, W.; Shu, S.; Liu, Z.; Liu, Y.; Kong, J.; Ma, E.; Cao, Y.; Liu, R.-S., Highly efficient non-rare-earth red emitting phosphor for warm white light-emitting diodes. *Nat. Comm.* **2014,** *5*, 4312.

2. Leatherdale, C. A.; Woo, W.-K.; Mikulec, F. V.; Bawendi, M. G., On the absorption cross section of CdSe nanocrystal quantum dots. *The J. of Phys. Chem. B* **2002,** *106* (31), 7619-7622.

3. Liu, Z.; Bekenstein, Y.; Ye, X.; Nguyen, S. C.; Swabeck, J.; Zhang, D.; Lee, S.-T.; Yang, P.; Ma, W.; Alivisatos, A. P., Ligand Mediated Transformation of Cesium Lead Bromide Perovskite Nanocrystals to Lead Depleted Cs4PbBr6 Nanocrystals. *J. Am. Chem. Soc* **2017,** *139* (15), 5309-5312.

4. Rakhshani, A., Study of Urbach tail, bandgap energy and grain-boundary characteristics in CdS by modulated photocurrent spectroscopy. *J. Phys.: Condens. Matter* **2000,** *12* (19), 4391.
